# Fibroblast growth factor receptor 3-IIIc mediates colorectal cancer growth and migration

**DOI:** 10.1038/sj.bjc.6605596

**Published:** 2010-03-16

**Authors:** G Sonvilla, S Allerstorfer, C Heinzle, S Stättner, J Karner, M Klimpfinger, F Wrba, H Fischer, C Gauglhofer, S Spiegl-Kreinecker, B Grasl-Kraupp, K Holzmann, M Grusch, W Berger, B Marian

**Affiliations:** 1 Department of Medicine 1, Institute of Cancer Research, Medical University Vienna, Vienna, Austria; 2 Social Medical Centre South, Vienna, Austria; 3 Institute of Clinical Pathology, Medical University Vienna, Vienna, Austria; 4 Department of Neurosurgery, Landesnervenklinik Wagner-Jauregg Hospital, Linz, Austria

**Keywords:** colon cancer, FGFR3, therapeutic target, clonogenicity, cell migration

## Abstract

**Background:**

Deregulation of fibroblast growth factor receptor 3 (FGFR3) is involved in several malignancies. Its role in colorectal cancer has not been assessed before.

**Methods:**

Expression of FGFR3 in human colorectal tumour specimens was analysed using splice variant-specific real-time reverse transcriptase PCR assays. To analyse the impact of FGFR3-IIIc expression on tumour cell biology, colon cancer cell models overexpressing wild-type (WT-3b and WT3c) or dominant-negative FGFR3 variants (KD3c and KD3b) were generated by either plasmid transfection or adenoviral transduction.

**Results:**

Although FGFR3 mRNA expression is downregulated in colorectal cancer, alterations mainly affected the FGFR3-IIIb splice variant, resulting in an increased IIIc/IIIb ratio predominantly in a subgroup of advanced tumours. Overexpression of WT3c increased proliferation, survival and colony formation in all colon cancer cell models tested, whereas WT3b had little activity. In addition, it conferred sensitivity to autocrine FGF18-mediated growth and migration signals in SW480 cells with low endogenous FGFR3-IIIc expression. Disruption of FGFR3-IIIc-dependent signalling by dominant-negative FGFR3-IIIc or small interfering RNA-mediated FGFR3-IIIc knockdown resulted in inhibition of cell growth and induction of apoptosis, which could not be observed when FGFR3-IIIb was blocked. In addition, KD3c expression blocked colony formation and migration and distinctly attenuated tumour growth in SCID mouse xenograft models.

**Conclusion:**

Our data show that FGFR3-IIIc exerts oncogenic functions by mediating FGF18 effects in colorectal cancer and may constitute a promising new target for therapeutic interventions.

Pathogenesis of cancer results from progressive deregulation of cell homeostasis. It affects proliferation and cell death, differentiation, cell migration and tissue interactions (Hanahan *et al*, 1996) by deregulating intracellular signalling pathways. Such deregulation frequently involves receptor tyrosine kinases – a fact that makes those receptors suitable targets for cancer therapy (Zwick *et al*, 2001). Fibroblast growth factors (FGFs) and their tyrosine kinase receptors (FGFR) have significant roles in development, wound healing and angiogenesis. Consequently, their deregulation can contribute to tumour development at several levels by stimulating proliferation, survival, cell migration and vascularisation of tumours (Eswarakumar *et al*, 2005).

The human genome contains 22 genes coding for FGFs and four genes coding for functional FGFRs. The receptors in general possess an extracellular ligand-binding domain consisting of three immunoglobulin (Ig)-like loops, a short transmembrane domain and an intracellular kinase domain. Differential splicing creates multiple variants lacking IgG loops, the transmembrane domain or the cytoplasmic domain (Powers *et al*, 2000). Ligand binding occurs in Ig-like loop III, in which alternative splice variants result in IIIb and IIIc isoforms of FGFR1–3, with different ligand specificities (Powers *et al*, 2000; Ornitz and Itoh, 2001). IIIb forms are frequently expressed in epithelial tissues, whereas IIIc forms are observed mainly in the mesenchyme (Orr-Urtreger *et al*, 1993; Murgue *et al*, 1994; Scotet and Houssaint, 1995), where they mediate tissue interactions in normal development and wound healing, together with ligands expressed in adjacent tissues. Carcinomas have been observed to switch expression of FGFR to the mesenchymal isoform, enabling cells to receive signals usually restricted to the connective tissue, leading to more aggressive phenotypes (Yan *et al*, 1993; Carstens *et al*, 1997).

Fibroblast growth factor receptor 3 (FGFR3) has been described as a negative regulator of bone growth (L’Hote and Knowles, 2005) and is also involved in carcinogenesis. Epithelial cells of the pancreas, kidney or lung express the splice-variant FGFR3-IIIb, whereas splice-variant IIIc has been found, for example, in chondrocytes (Murgue *et al*, 1994; Scotet and Houssaint, 1995; Wuechner *et al*, 1996; L’Hote and Knowles, 2005). In the intestinal epithelium of mice, both splice variants of FGFR3 are expressed in undifferentiated crypt cells, suggesting a role of FGFR3-IIIc in cell proliferation (Vidrich *et al*, 2004). In healthy epithelium, FGFR3 expression is regulated by FGF7 and transforming growth factor *β*, which are mediators of mucosal healing (Kanai *et al*, 1997a, 1997b).

In malignant cells, FGFR3 can be deregulated by three different mechanisms: activating mutations, differential expression and alterations in the predominant splice variant. Activating mutations in FGFR3 have been observed both in the ligand-binding and kinase domains in multiple myelomas, as well as in cancers of the bladder and cervix (L’Hote and Knowles, 2005). They are, however, rare in colorectal tumours (Jang *et al*, 2000). Here, downregulation of FGFR3 expression by a mechanism involving FGFR1 and differential splicing has been observed (Jang *et al*, 2000; Jang, 2005). No information is available yet with regard to the role of the IIIc splice variant of FGFR3 in colorectal tumours. This receptor may have a major impact on tumour cell characteristics because of its broad ligand spectrum (FGFs 1, 2, 4, 6, 9 and 18) compared with FGFR3-IIIb (FGFs 1 and 9 exclusively) (Kanai *et al*, 1997a). Consequently, a switch to IIIc as the main FGFR3 isoform may permit stimulation of tumour cells by those additional factors (Pandit *et al*, 2002; Davidson *et al*, 2005).

Among the FGFR3 ligands, FGF18 is upregulated in colon tumourigenesis (Shimokawa *et al*, 2003), allowing autocrine growth stimulation through FGFR3-IIIc. Accordingly, we have recently demonstrated strong autocrine effects of FGF18 on tumour cell growth and survival (Sonvilla *et al*, 2008). To investigate the role of FGFR3-IIIc in the pathogenesis of colorectal tumours, we have analysed expression of IIIb and IIIc variants in colorectal tumour tissue and examined the impact of FGFR3-IIIc signalling on growth, survival and migration of colorectal tumour cells *in vitro* and *in vivo*.

## Materials and methods

### Tissue specimen

Tissue specimens of colorectal carcinoma and normal mucosa were obtained from patients undergoing surgery for colorectal cancer after obtaining informed consent from all patients. Immediately after surgery, tissue specimens were frozen in liquid N_2_ until extraction of RNA. The tissue specimen has a tumour cell content of at least 70%, as judged from the histology of immediately adjacent tissue. The study had previous approval of the ethics committee of the Municipal Hospitals of Vienna.

### Cell lines

SW480, Caco2 and HCT116 colon carcinoma cell lines were obtained from the American Type Culture Collection. The cell lines were kept under standard tissue culture conditions using Minimal Essential Medium containing 10% foetal calf serum (FCS). The Vaco235 colon adenoma cell line was a gift from JKV Willson (Ireland Cancer Centre, Case Western University, Cleveland, OH, USA) and was maintained as described in the study by Willson *et al* (1987). LT97 adenoma cells were maintained as described by Richter *et al*, (2002). AKH14 and H64 colon carcinoma cell lines were established at our institute from a liver and brain metastasis, respectively, and cultivated in RPMI with 10% FCS.

### Isolation of RNA and cDNA synthesis

Total RNA was isolated from subconfluent cultures or frozen colon tissue specimens using Trifast reagent according to the manufacturer's instructions (PeqLab, Erlangen, Germany). First-strand cDNA was synthesised using RevertAid mouse Moloney leukemia virus (MMuLV) reverse transcriptase (Fermentas, Burlington, Ontario, Canada) and random hexamer primers (GE Healthcare, Piscataway, NJ, USA).

### Quantitative RT–PCR analysis

Taqman assays from Applied Biosystems (Foster City, CA, USA) were used to determine expression of total FGFR3 (Hs00179829), FGFR3-IIIc (Hs00997397-m1) and glyceraldehyde 3-phosphate dehydrogenase (GAPDH) (Hs 99999905-m1) mRNAs using quantitative reverse transcriptase PCR (RT–PCR). Probes specific for FGFR3-IIIb were custom designed by Applied Biosystems to recognise the exon 6/7 boundary specific for IIIb splice variants. Reactions were carried out on an ABI PRISM 7000 system (Applied Biosystems) using standard Taqman assay conditions. The FGFR3 gene expression in the cells and tumours was calculated as x-fold change compared with the corresponding normal mucosa using GAPDH as the control gene for normalisation using a standard curve method. In addition, selected RT–PCR experiments were performed using cycles of 40 s denaturation at 94°C, 40 s annealing at 56°C and 40 s extension at 72°C. Amplifications were carried out for 40 and 23 cycles in case of FGFR and GAPDH, respectively. PCR products were separated on 6% acrylamide gels. Bands were stained with ethidium bromide and quantified using a GelDoc system (Biorad, Hercules, CA, USA) and Image Quant 5.0 (GE Healthcare) software.

### Overexpression of FGFR in colon carcinoma cells

Stable transfectants of SW480 and HCT116 cells were prepared with plasmids expressing wild-type FGFR3-IIIc (WT3c) or kinase-dead FGFR3-IIIc (KD3c, mutation K508R) driven by a CMV promoter (plasmids kindly provided by DJ Donoghue; University of California, San Diego, CA, USA). The wild-type and kinase-dead FGFR3-IIIb constructs (WT3b, KD3b) were constructed by removing the exon 8 sequence in the respective FGFRIII-3c vectors and substituting it with exon 7. For that purpose, the respective sequence was amplified with primers forward 5′-CTGCGTCGTGGAGAACAAG-3′ and reverse 5′-CCGAGACAGCTCCCATTTG-3′ and Pfu proofreading polymerase (Stratagene, La Jolla, CA, USA) from cDNA of Vaco235 cells, which predominantly express the IIIb form. The PCR product was digested with *Eco*47III and *Xho*I, and ligated into the similarly digested KD3c plasmid. The resulting KD3b plasmid was checked by sequencing. Cells that overexpressed FGFR3 were produced through transfection by electroporation. Controls received equal amounts of vector DNA (pcDNA3, Invitrogen, Lofer, Austria). Stably overexpressing cells were obtained by selection with 0.6 mg ml^–1^ geneticin (G418; PAA, Pasching, Austria).

The dominant-negative FGFR3-IIIc adenoviral construct KD3-IIIcv was prepared using the KD3c plasmid and the AdEasy adenoviral system (Stratagene), following the manufacturer's instructions. As controls, adenoviral constructs expressing GFP (GFPv) or a scrambled sequence (control virus, Cv) were used, all controlled by a CMV promoter. Cells were seeded at a density of 2 × 10^5^ in six-well plates in medium containing 10% FCS. Subsequently, they were infected at a multiplicity of infection of 1 for SW480 cells and 20 for Caco2 cells, yielding >90% of green fluorescent cells with the GFP virus.

### Knockdown of gene expression

Small interfering RNAs (siRNAs) specifically targeting the IIIc-specific exon 8 or the IIIb-specific exon 7, but not the respective other exon, were custom designed by Ambion (Applied Biosystems). Cells were seeded at a density of 2 × 10^5^ cells per well into six-well plates in medium containing 10% serum. After 24 h, they were transfected with 3 *μ*l Silentfect (BioRad) and 20 pmol of siRNA per well in culture medium without serum. A scrambled siRNA without sequence homology to any known human gene and siRNA directed against GAPDH served as negative controls. After 24 and 48 h, RNA and protein were isolated to verify knockdown efficiency.

### Protein isolation and western blotting

Total protein extraction and western blotting were performed as described (Sonvilla *et al*, 2008) using the following antibodies: FGFR3 (C-15) (#sc-123, recognises both FGFR3 splice variants; Santa Cruz Biotechnology, Inc., CA, USA), phospho-S6 (#2215) and S6 Ribosomal Protein 1 : 1000 (#2212, Cell Signalling, Boston, MA, USA), phospho-externally regulated kinase (ERK)1/2 (sampler kit: phospho-p44/42 MAP Kinase Thr202/Tyr204, Cell Signalling), ERK1/2 1 : 5000 (#06-182, Upstate, Lake Placid, NY, USA), caspase 3 1 : 1000 (H277, Santa Cruz Biotechnology, Inc.) and *β*-actin 1 : 5000 (AC-15, SIGMA, Saint Louis, MO, USA).

### Cell viability and cell proliferation assays

Cells were seeded at a density of 3 × 10^3^ cells per well into 96-well plates. For knockdown experiments, cells were transfected with siRNA directed against FGFR3 after 24 and 48 h, and viability was determined after an additional 48 h recovery period. For growth stimulation with recombinant growth factors, cells were serum starved for 24 h and then stimulated with recombinant FGF18 (hFGF18-25, Strathmann Biotec, Bovenau, Germany) for 4 days. Cell viability was determined by 3′ (4,5-dimethylthiazol-2-yl)-2,5-diphenyltetrazoliumbromide (MTT) assay (EZ4U; Biomedica, Vienna, Austria). For determination of DNA synthesis, cells were incubated with ^3^H-thymidine (1 *μ*Ci ml^–1^) for 1 h, washed twice with phosphate-buffered saline and processed for scintillation counting.

### Colony formation assay

SW480 and HCT116 cells were plated at a density of 100 or 200 cells per well, respectively, onto six-well plates in medium containing 10% FCS. After 10 days, the cells were washed with phosphate-buffered saline and fixed with methanol for 20 min. After washing with phosphate-buffered saline, cells were stained with 0.01% of crystal violet solution to assess colony formation.

### Cell migration assay

Confluent monolayers of different cell lines were serum starved for 24 h and ‘scratch’-wounded using a yellow pipette tip. At each time point, four defined parts of each scratch were photographed and individual scratch widths (*μ*m, mean±s.d.) were measured using MetaMorph software (Meta Imaging Series 6.1, Universal Imaging Corporation, Dowingtown, PA, USA). Migration distance was calculated from the difference of the scratch width.

For SW480 cells transfected with siRNA, as well as SW480 and Caco2 cells infected with adenoviral constructs expressing dominant-negative FGFRs, migration assays were performed using 8-*μ*m-pore-sized PET track-etched membrane (BD-Falcon, Franklin Lakes, NJ, USA) in 24-well plates. Cells were seeded at a density of 0.5 × 10^5^ cells cm^–2^. After a migration period of 24 and 48 h for SW480 and Caco2 cells, respectively, the nitrocellulose filters were removed and cells at the bottom of the membrane and in the lower chamber were fixed and stained with crystal violet. The number of cells or colonies was counted microscopically.

### Tumour growth

SW480 cells were infected with 1 multiplicity of infection KD3-IIIcv or GFPv on cell culture plates. After 24 h, cells were harvested, washed with phosphate-buffered saline and suspended in Ringer's solution. In all, 1 × 10^6^ cells in 50 *μ*l Ringer's solution were subcutaneously injected into the rear flanks of immunodeficient SCID/Balb/c recipient mice (female, aged 4 weeks, Harlan Winkelmann, Borchen, Germany). Tumour formation was monitored periodically by palpation, and tumour size was determined using a vernier caliper. Tumour volume was calculated using the formula (smaller diameter^2^ × larger diameter)/2. All experiments were performed in triplicate and carried out according to the Austrian and FELASA guidelines for animal care and protection. Tissue sections of experimental tumours were analysed using immunohistochemistry as previously described (Berger *et al*, 2005). Proliferation and apoptosis were evaluated by counting Ki67-positive cells and apoptotic structures, respectively, from 3 to 5 random areas of each tumour section and calculated as a percentage of total cells.

### Cell death detection and caspase activity

Cell death was determined by staining for 30 min with 5 *μ*g ml^–1^ Hoechst 33258 and 2 *μ*g ml^–1^ propidium iodide 5 days after siRNA transfection or adenoviral transduction. Alternatively, for determination of caspase activity, the medium was replaced by 50 *μ*l rhodamine-labelled caspase substrate D_2_R (Alexis Biochemicals, Lausen, CH, USA) and cells were incubated for 30 min at 37°C. The fluorescent rhodamine cleavage product is released into the culture supernatant and measured in the fluorescein isothiocyanate channel at *λ*ex488/*λ*em550 nm on a fluorometer (Synergy HT, Multi-Detection Microplate Reader, BIO-TEK Instruments, Inc., Winooski, VT, USA).

### Statistical analysis

Tissue expression data were analysed by analysis of variance after obtaining a Gaussian distribution by transforming to log values. The relationship between tumour stage and shift in the FGFR3 IIIc/IIIb ratio was determined in comparison with stage I using contingency tables and Fisher's exact test. All cell biological results were analysed by Student's *t*-test or Kruskal–Wallis test, depending on the results of normality testing.

## Results

### Expression of FGFR3 in colon carcinoma tumour tissues

**Figure 1:**
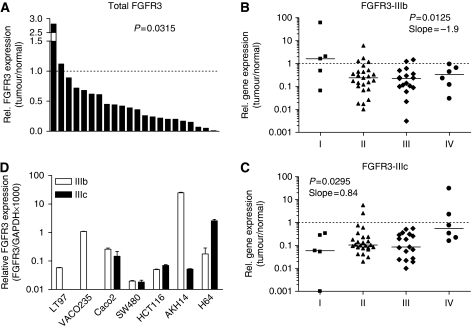
Expression of fibroblast growth factor receptor 3 (FGFR3) mRNA in colorectal cancer. RNA was isolated from surgical specimens of colorectal tumours and adjacent normal mucosa and expression of FGFR3 was quantified by reverse transcriptase PCR (RT–PCR). Expression levels were calculated relative to glyceraldehyde 3-phosphate dehydrogenase (GAPDH), normalised relative to expression in normal mucosa. Repeated assays from each specimen yielded cycle threshold (*C*_t_)-values <0.5 cycles apart. (**A**) Total FGFR; (**B**) FGFR3-IIIb; and (**C**) FGFR3-IIIc. (**B** and **C**) Results are grouped by stage of disease and each column shows the individual values with the mean of expression marked by a horizontal line. Differences between tumour and adjacent normal mucosa were analysed by analysis of variance from the log values of expression. *P*-values and linear trend are given in the inserts. (**D**) The FGFR3 splice-variant pattern expressed in colorectal tumour cell lines was quantified by real-time RT–PCR (right panel, mean±s.d. of three independent experiments).

Total RNA was isolated from normal and tumour-derived tissues (*N*=22) and expression of FGFR3 mRNA was measured by quantitative real-time RT–PCR relative to GAPDH. Expression of total FGFR3 mRNA was found to be downregulated in 20 of 22 tumour specimens, resulting in a significant overall reduction in tumours compared with non-malignant colon tissue specimens (Figure 1A, *P*=0.0015). Expression of FGFR3 isoforms IIIb and IIIc was determined for a larger number of tumour specimens (*n*=57) using isoform-specific real-time RT–PCR assays. The FGFR3-IIIb was downregulated in 49 of these specimens. Expression of FGFR3-IIIb was downregulated with increasing stage (Figure 1B; *P*=0.0125, slope= −1.9). By contrast, FGFR3-IIIc expression increased with stage (Figure 1C; *P*=0.0303, slope= +0.84). As a consequence, the IIIb/IIIc ratio, compared with normal mucosa, increased with stage at a slope of +2.622 (*P*=0.0023). The fraction of tumours with a higher IIIc/IIIb ratio also significantly increased with higher stages (Table 1).

Colorectal carcinoma cell lines also expressed the IIIb and IIIc variants of FGFR3 at different ratios, clearly showing FGFR3-IIIc expression in the epithelial cell compartment. No FGFR3-IIIc was detected in the adenoma cell lines, whereas the highest IIIc/IIIb ratio was found in the H64 brain metastasis-derived cells (Figure 1D).

### Impact of FGFR3-IIIc on cell growth and clonogenicity

SW480 and HCT116 cells were used to create cell line models stably overexpressing FGFR3. From the slowly growing Caco2 cells, stable transfectants could not be obtained. Plasmids expressing either wild-type FGFR3-IIIb or -IIIc receptors (WT3b and WT3c) or kinase-dead dominant-negative mutants of both FGFR3-IIIb and FGFR3-IIIc (KD3b and KD3c) were introduced by electroporation and stable transfectants were selected. Overexpression of FGFR3 in pools of stable clones was checked by RT–PCR using IIIb- or IIIc-specific primers on mRNA and with western blot analysis on protein level. In IIIc transfectants, the receptor subtype was found to be 15- to 20-fold at the RNA level, whereas expression of the IIIb variant was not affected. In IIIb transfectants, overexpression was >100-fold for IIIb at the RNA level. A distinct increase in total FGFR3 protein was found in all transfectants (for details see Supplementary Material). Transient expression of dominant-negative FGFR3 was achieved from an adenoviral construct expressing KD3 (KD3-IIIcv). A GFP-tagged virus (GFPv), an untagged Cv and uninfected parental SW480 cells were used for comparison. Splice-variant IIIc mRNA was specifically overexpressed up to 20-fold (Supplementary Material).

**Figure 2:**
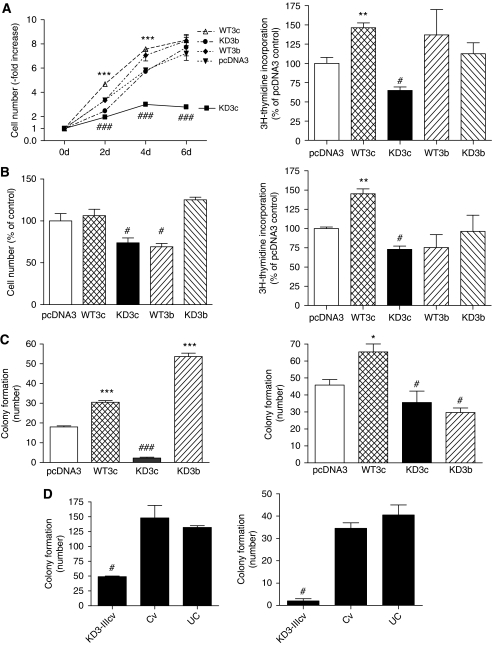
Impact of fibroblast growth factor receptor 3-IIIc (FGFR3-IIIc) on growth and clonogenicity. (**A**) SW480 and (**B**) HCT116 transfectants were plated at 3 × 10^3^ cells per well in 96-well plates and growth was determined by 3′ (4,5-dimethylthiazol-2-yl)-2,5-diphenyltetrazoliumbromide (MTT) assay at the indicated time points (**A**) or after 3 days (**B**). Data points represent the mean±s.e.m. of 10 determinations from two independent experiments (left panels). In all, 2.5 × 10^4^ cells were plated for assessment of DNA synthesis. After 24 h, cultures were incubated with 1 *μ*Ci  ml^–1 3^H-thymidine for 1 h and incorporation of radioactivity into DNA was determined as described in Sonvilla *et al* (2008) (right panels). (**C**) Stable SW480 and HCT116 transfectants were plated at 100 and 200 cells per well, respectively, in six-well plates, fixed and stained with crystal violet 10 days later to show colony formation. Colonies were counted from two experiments using duplicate cultures (means±s.e.m.). SW480 (left panel), HCT116 (right panel). (**D**) SW480 and Caco2 cells were infected with kinase-dead FGFR3 (KD3)-IIIcv or control virus (Cv). Cloning efficiency was determined from 200 cells per well and compared with untransduced control (UC). ^*^, ^**^, ^***^ and #, ### indicate increase or a decrease, respectively, as compared with vector controls at *P*<0.05, *P*<0.01 or *P*<0.0001 (Student's *t*-test). d, day.

To determine the impact of the different FGFR3 constructs on cell growth, stable SW480 transfectants were seeded at equal density and kept in medium containing 10% FCS for up to 6 days. Cell viability was determined at the indicated time points. Cells expressing WT3c grew faster than control transfectants, reaching similar density after 6 days. Growth of WT3b cells was also increased, compared with control transfectants, but the effect was smaller than for WT3c and only just significant. KD3b did not affect cell growth in SW480 cells, but KD3c cultures showed decreased growth at all time points (Figure 2A, left panel). This effect became more severe with time in culture and KD3c transfectants lost viability after 3–4 passages. By contrast, vigorously growing cultures, the growth characteristics of which were identical to control transfectants, could be isolated from SW480-KD3b transfectants. DNA synthesis was measured by ^3^H-thymidine incorporation assays 24 h after plating. DNA synthesis was stimulated by WT3c but not by WT3b. Inhibition was observed in KD3c overexpressing cells, whereas KD3b again had no impact (Figure 2A, right panel). Stable HCT116 transfectants overexpressing FGFR3-IIIc were less sensitive to the impact of FGFR3-IIIc overexpression and was inhibited by FGFR3-IIIb overexpression (Figure 2B). Cell viability after 3 days in culture was not affected by the WT3c or KD3b construct, but was significantly decreased by WT3b and KD3c (70% of control). ^3^H-thymidine incorporation was stimulated by WT3c, reduced by WT3b and KD3c, and not affected by KD3b (Figure 2B right panel).

Differences in growth potential became even more obvious under the stringent conditions of colony formation assays. SW480 cells were plated at 100 cells per well and colony formation was determined after 10 days. Colony formation was increased about two-fold in WT3c and almost completely suppressed in KD3c transfectants. By contrast, KD3b increased plating of SW480 cells (Figure 2C, left panel). Colony formation of HCT116 cells was also enhanced by overexpression of WT3c, but the cells were less sensitive to inhibition by KD3c. KD3b caused a decrease in plating (Figure 2C, right panel). Impact of the dominant-negative construct was also assessed by transduction with an adenoviral vector in SW480 and Caco2 cells. Cv was used as control and stimulated the clonogenicity of SW480 but not of Caco2 cells, compared with uninfected cells (UC). KD3-IIIc virus severely inhibited attachment in both cell lines (Figure 2D).

### Induction of apoptosis by blocking FGFR3-IIIc signalling

**Figure 3:**
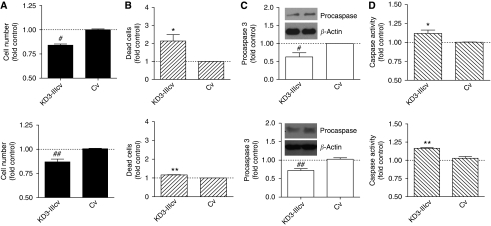
Induction of apoptosis by dominant-negative fibroblast growth factor receptor 3 (FGFR3). SW480 (upper panels) and Caco2 (lower panels) cells were infected with adenoviral constructs expressing kinase-dead FGFR3-IIIc (KD3-IIIcv) and control virus (Cv) at multiplicity of infections (MOIs) of 1 (SW480) or 20 (Caco2). (**A**) Cell viability was determined 6 days later by 3′ (4,5-dimethylthiazol-2-yl)-2,5-diphenyltetrazoliumbromide (MTT) assay. The data points represent the mean±s.e.m. of two independent experiments using quadruplet cultures. (**B**) Apoptotic cells were detected after staining with Hoechst dye and propidium iodide to visualise nuclear morphology and counted from 1000 cells, each in triplicate cultures. (**C**) To determine caspase activation, cell lysates were analysed by western blot using a monoclonal antibody recognising procaspase 3. Inserts depict representative blots. Quantification of band intensities was performed from three independent experiments. (**D**) Caspase activity was determined using a substrate predominantly cleaved by caspase 3. The data points represent the mean±s.e.m. of two independent experiments using quadruplet cultures. ^*^, ^**^Indicate increase as compared with Cv at *P*<0.05 and *P*<0.01, respectively. #, ## indicate decrease as compared with Cv at *P*<0.05 and *P*<0.01 (Student's *t*-test).

The impact of KD3-IIIcv transduction on cell survival was assessed and compared with Cv in SW480 (Figure 3; upper panels) and Caco2 cells (Figure 3; lower panels). Multiplicity of infections of 1 and 20 were used for transduction of SW480 and Caco2 cells, respectively. After 6 days, KD3-IIIcv reduced cell viability in both cell lines (Figure 3A). In SW480 cell cultures, a 17% cell loss was observed and the incidence of apoptotic cells was doubled. In Caco2 cultures, a similar cell loss (14%) was accompanied by an increase of only 15% in the incidence of apoptosis (Figure 3B). Although this effect was relatively minor, it was also highly reproducible and significant at *P*<0.01. In addition, protein lysates were obtained on day 6 and procaspase 3 was analysed by western blotting (Figure 3C). The amount of procaspase was decreased in the KD3-IIIcv groups of both cell lines, indicating cleavage to produce active caspase 3 (Figure 3C). Accordingly, caspase activity increased in parallel (Figure 3D).

**Figure 4:**
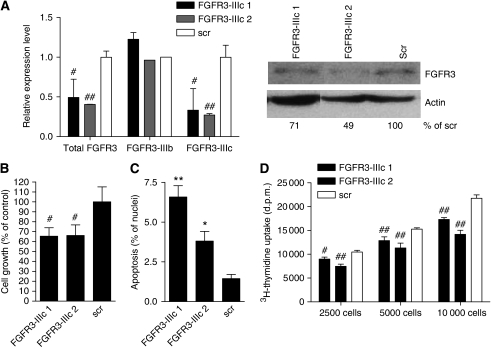
Impact of fibroblast growth factor receptor 3c (FGFR3c) knockdown on growth and viability. (**A**) Small interfering RNA (siRNA) directed against FGFR3-IIIc was introduced into SW480 cells by lipofection, and RNA (left panel) and protein (right panel) were assessed 48 h later. (**B**–**D**) For determination of growth and viability, cells were plated in 96-well plates at 2 × 10^3^ cells per well and siRNA was delivered twice 24 and 48 h later. After 48 h, cell viability (**B**) and induction of apoptosis (**C**) and DNA synthesis (**D**) were determined as described in the Materials and methods section. ^*^, ^**^ and #, ## indicate increase or decrease, respectively, as compared with control at *P*<0.05 or *P*<0.01 (Student's *t*-test).

**Figure 5:**
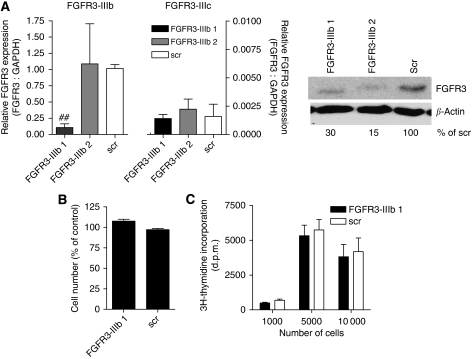
Impact of fibroblast growth factor receptor 3b (FGFR3b) knockdown on growth and viability. (**A**) Small interfering RNA (siRNA) directed against FGFR3-IIIb was introduced into HCT116 cells by lipofection, and RNA (left panel) and protein level (right panel) were assessed 48 h later. (**B**) For determination of growth and viability, cells were plated in two-well plates at 2 × 10^3^ cells per well and siRNA was delivered 48 h before cell viability was determined as described in the Materials and methods section. (**C**) DNA synthesis was measured by ^3^H-thymidine incorporation. All data represent the pooled results of at least three independent experiments and no statistically significant effect could be detected. ## indicates a decrease as compared with control at *P*<0.05 (Student's *t*-test).

In addition, FGFR3-IIIc expression in SW480 cells was knocked down using siRNA that caused a reduction of both mRNA and protein levels (Figure 4A). The siRNA was most effective after double transfection 24 and 48 h after plating, reducing FGFR3-IIIc mRNA by 75%, but only minimally affecting FGFR3-IIIb (Figure 4A, left panel). The protein level was similarly reduced by 30 and 50%, respectively (Figure 4A, right panel). Cell viability was assessed another 48 h later and found to be reduced by 35% as compared with the scrambled control (Figure 4B). At the same time, incidence of apoptosis was increased 4.7- and 2.7-fold, respectively (Figure 4C). ^3^H-thymidine incorporation was inhibited at all cell densities, but was strongest in dense cultures (Figure 4D; 80 and 65% of control). To control for the subtype specificity of the effects, a similar series of knockdown experiments was performed for FGFR3-IIIb: siFGFR3-IIIb-1 caused a 90% reduction of FGFR3-IIIb mRNA and a 70% reduction of FGFR3 protein, but no alteration of FGFR3-IIIc expression. Knockdown of siFGFR3-IIIb2 was unreliable and was therefore not included in the growth assays (Figure 5A). Downmodulation of FGFR3-IIIb did not result in either a reduction of viable cells in the culture or in a decrease of DNA synthesis (Figure 5B).

### Impact of FGFR3-IIIc blockade on tumour growth *in vivo*

**Figure 6:**
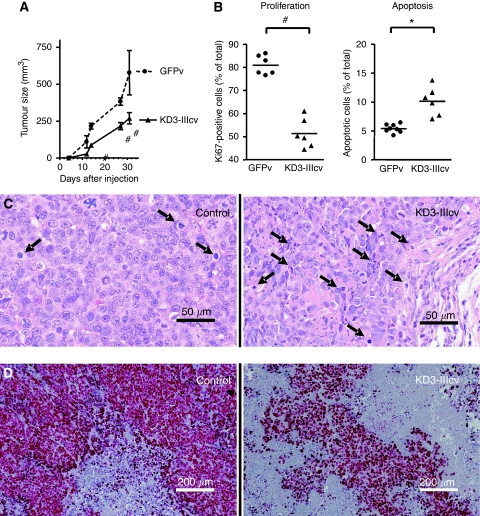
Impact of fibroblast growth factor receptor 3 (FGFR3) blockade on tumour growth *in vivo.* SW480 cells were infected with kinase-dead FGFR3 (KD3)-IIIcv or green fluorescent protein-tagged virus (GFPv) and then injected subcutaneously into SCID mice. Tumour growth was monitored (**A**) and tumour tissue processed for histopathological examination. Paraffin-embedded tissue sections were stained with haematoxilin–eosin (**C**) and with an antibody recognising the proliferation marker Ki67 (**D**). Arrows point at apoptotic structures. The fraction of Ki67-positive cells and that of apoptotic cells were counted from 3 to 5 random fields of vision per section (**B**). ^*^ and # indicate an increase or decrease as compared with control at *P*<0.05 (Student's *t*-test).

As *in vitro* growth assays strongly indicated an anti-tumourigenic effect of FGFR3 blockade, tumour growth was assessed *in vivo* by injection of KD3-IIIcv- and GFPv-transduced SW480 cells into SCID mice. Tumour growth was significantly retarded by FGFR3 blockade as compared with GFPv-infected controls (Figure 6A) and uninfected cells (data not shown). Tumour tissues were analysed by staining paraffin-embedded sections with either haematoxylin–eosin (Figure 6C) or antibodies recognising Ki67 (Figure 6D). Because of their larger size, GFP tumours contained significantly larger areas of necrotic tissue. Analysis of growth parameters was therefore restricted to non-necrotic tumour regions. In these viable tumour areas, the fraction of Ki67-positive cells was significantly decreased in KD3-IIIc tumours (Figure 6B, left panel), although incidence of apoptosis was increased (Figure 6B, right panel), compared with control tumours.

### Transduction of ligand-dependent signals

**Figure 7:**
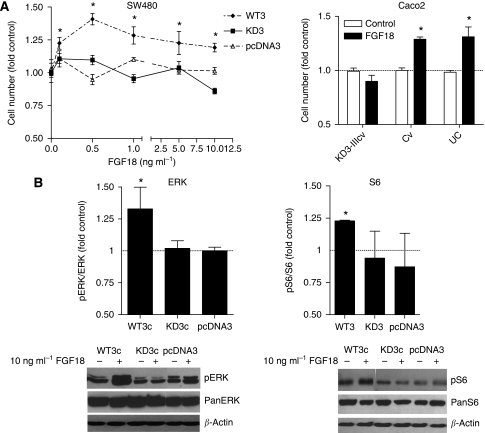
Fibroblast growth factor receptor 3-IIIc (FGFR3-IIIc)-dependent growth signalling and cell migration. (**A**) To determine FGFR3-IIIc dependency of cell signalling, SW480 transfectants were stimulated by FGF18 after 2 days of serum starvation (left panel). Alternatively, Caco2 cells were infected with kinase-dead FGFR3 (KD3)-IIIcv 2 days before addition of FGF18 (10 ng ml^–1^; right panel). Data points represent the mean±s.e.m. from two experiments using quintuplet cultures. (**B**) FGFR3 transfectants were exposed to FGF18 (10 ng ml^–1^) after 2 days of serum starvation. Protein lysates were obtained 15 min later and phosphorylation of externally regulated kinase (ERK) and that of S6 were determined by western blotting using phosphorylation-specific antibodies. Band intensities were determined from three independent experiments by Image Quant software and phosphorylation was calculated relative to total protein. ^*^Indicates an increase compared with control at *P*<0.05 (Kruskal–Wallis test).

Previous experiments had shown that exogenous FGF18 did not stimulate growth under standard conditions in FGFR3-low SW480 cells (Sonvilla *et al*, 2008). To determine whether FGFR3-IIIc can confer FGF18 sensitivity to cells, receptor transfectants were kept in serum-free culture medium for 2 days before stimulation with recombinant FGF18 (Figure 7A). After 4 days , the cell number was measured by MTT assay and demonstrated dose-dependent growth stimulation in WT3c cells. KD3c cells were not stimulated (Figure 7A, left panel). As the data suggest that FGFR3-IIIc confers FGF18 responsiveness, the impact of FGFR3 inhibition on FGF18 sensitivity was determined by infecting cells with the dominant-negative KD3-IIIcv and Cv before exposure to the growth factor. This had no effect on FGF18-insenstitive SW480 cells (data not shown). On the other hand, FGFR3-IIIc-high Caco2 cells responded to FGF18 with a 25% increase in cell viability when infected with control viruses, and KD3-IIIcv completely abolished the response (Figure 7A, right panel).

To investigate the impact of FGFR3-IIIc on FGF18-dependent downstream signalling, stable SW480-FGFR3 transfectants were serum starved for 24 h, stimulated with 10 ng ml^–1^ recombinant FGF18 and, 15 min later, phosphorylation of ERK and S6 was determined by western blotting. Phosphorylation of both signalling molecules was stimulated by FGF18 only in WT3 cells to 132±0.3% for ERK and to 123±0.01% for S6. In KD3c cultures, control levels of ERK phosphorylation were lower than in controls or in WT3c transfectants, and the growth factor did not lead to pathway stimulation (Figure 7B).

### Impact of FGFR3-IIIc on cell migration

**Figure 8:**
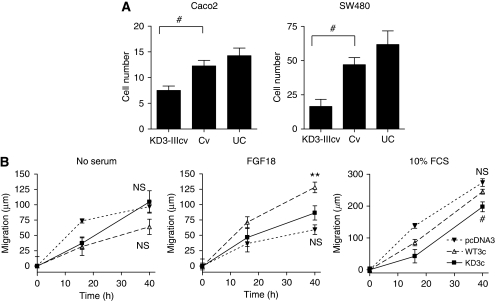
Fibroblast growth factor receptor 3-IIIc (FGFR3-IIIc)-dependent tumour cell migration. (**A**) FGFR3-IIIc-dependent signalling was inhibited by infection with dominant-negative adenoviral constructs in SW480 (left panel) and Caco2 cells (right panel). Cells were seeded into filter inserts at 0.5 × 10^5^ cells per well and kept in 1% serum for 24 h. Cell migration was determined by staining cells at the bottom of the membrane or in the lower chamber with crystal violet. (**B**) Stable FGFR3 transfectants were grown to confluence in six-well plates and scratch wounded. Migration was determined by measuring scratch closure over time. Data points represent the distance migrated by each culture. All data points are calculated as the mean±s.e.m. from four cultures, with ^**^ and # indicating an increase or decrease as compared with control at *P*<0.05 (Kruskal–Wallis test). NS, not significant.

To assess whether FGFR3-IIIc mediates migration signals, we performed migration assays with SW480 and Caco2 cells after infection with dominant-negative KD3-IIIcv and Cv. Uninfected cells and cells infected with Cv readily migrated through the filter, but the dominant-negative FGFR construct KD3c significantly reduced migration at comparable levels for both cell lines (Figure 8A).

In addition, we performed scratch assays with stably FGFR3-IIIc-transfected SW480 cells after 24 h serum starvation. Migration of cells was followed up for up to 40 h (doubling time >48 h). In starvation medium without serum, all transfectants migrated 60–100 *μ*m during the 40 h period without any significant differences between experimental groups (Figure 8B, left panel). Addition of 10 ng ml^–1^ FGF18 stimulated WT3c cells to migrate 128 *μ*m (*P*=0.0005), whereas the pcDNA3 and KD3c groups remained within the 60–100 *μ*m range (Figure 8B, center panel). A 10% FCS stimulated migration in all three groups. pcDNA3 and WT3c reached 274 and 246 *μ*m, respectively (Figure 8B, right panel; not significantly different *P*=0.052). KD3c inhibited migration as compared with the pcDNA3 control (Figure 8B, right panel; 197 *μ*m, *P*=0.01). However, KD3c cells were still stimulated by 10% serum (Figure 8B, left and right panels; *P*=0.013).

## Discussion

The FGFR3 is a tyrosine kinase receptor, the constitutive activation of which is involved in skeletal malformations (Bellus *et al*, 2000) and drives tumour cell growth in bladder and cervix cancer (Cappellen *et al*, 1999; Hernandez *et al*, 2006; Mhawech-Fauceglia *et al*, 2006). Deregulation of FGFR3 signalling may be caused by mutation, transcriptional regulation or differential splicing. The latter mechanism most prominently affects the Ig-like loop III, producing IIIb and IIIc variants, which are expressed preferentially in epithelia and connective tissue, respectively (Murgue *et al*, 1994). In colon carcinomas, receptor mutations are rare (Jang *et al*, 2000), but aberrant splicing causes a decrease in functional FGFR3-IIIb, accompanied by a switch to FGFR1-IIIc (Jang, 2005).

Our results now confirm the downregulation of FGFR3-III, and also demonstrate differential effects on the IIIb and IIIc variants. Specifically, expression of the IIIc splice variant can be retained better so that the IIIc/IIIb ratio is higher in tumour tissue than in normal mucosa. This was observed in about 50% of the tumour specimens analysed and preferentially in more advanced tumours. The IIIc splice variant was also detected in colon carcinoma, but not in adenoma-derived cell lines, demonstrating (1) that expression of the splice variant is not only due to mesenchymal contaminants but also comes from the epithelial compartment of the tumour and (2) that it is upregulated during tumour progression.

Switching of FGFR expression to the IIIc isotypes has been shown for FGFR2 and FGFR1, and was described to be associated with tumour progression in prostate (Yan *et al*, 1993; Carstens *et al*, 1997; Feng *et al*, 1997) and bladder cancer (Chaffer *et al*, 2006). For FGFR3, mutation of the IIIc variant causes tumour growth in multiple myeloma cells (Chesi *et al*, 2001). Our results now demonstrate that overexpression of FGFR3-IIIc stimulates tumour cell growth, whereas its blockade inhibits cell growth and induced apoptosis in colorectal cancer cells. Overexpression or blockade of FGFR3-IIIb had little impact on cell growth. Only in HCT116-KD3b transfectants was colony formation also decreased by blockade of IIIb; however, this effect was transient, and 3 or more days after plating, no evidence of growth inhibition by the construct remained. This is in agreement with the observation of Jang (2005) that overexpression of WT3b in the human HCT116 colorectal carcinoma cell line has been shown to inhibit cloning efficiency so that loss of expression in colorectal cancer *per se* should enhance growth. Expression of the FGFR3-IIIc splice variant as shown by our results is generally low, but seems to be essential for the growth and migration of colorectal tumour cells. In that manner, FGFR3-IIIc induced a distinctly different cellular response than the IIIb variant, which may be due to its larger range of ligands either by ligand-dependent heterodimerisation or through ligand-dependent differences in receptor downregulation and distribution to cellular compartments, as has also been described for the epidermal growth factor receptor (Resat *et al*, 2003). Results from *in vitro* models indicate that the IIIc splice variant is essential for the mediation of oncogenic FGF18 effects – such as colony formation, growth, survival and migration, which was stimulated by expression of the WT3c allele in SW480 cells and, to a lesser extent, in HCT116 cells. SW480 cells also acquired sensitivity to exogenous FGF18 after knockdown of endogenous factor production (Shimokawa *et al*, 2003; Sonvilla *et al*, 2008), suggesting that insensitivity was caused by autocrine growth factor saturating the available receptors (Berger *et al*, 1999; Barbieri *et al*, 2004; Reilly *et al*, 2004). Transfection of SW480 cells with WT3c had the same effect, probably by providing additional receptor binding sites. Transfectants responded by increased cell viability, migration and activation of intracellular signalling pathways. However, FGFR3-IIIc not only exclusively bind FGF18, but also FGFs 1, 2, 4, 8 and 9, among which FGF9 is expressed in colorectal tumour cells (data not shown). Similar to FGF18, this FGF also stimulated cell growth when added to WT3c transfectants, but did not affect controls, indicating another possibility of autocrine growth stimulation (data not shown). An oncogenic effect of FGFR3-IIIc is further supported by the inhibition of colony formation and induction of apoptosis by KD3c in all cell lines used and by the inhibition of FGF18 signals in Caco2 cells.

Accumulating data suggest that FGFR3 is a therapeutic target in both multiple myeloma and bladder cancer. Inhibition of this receptor by either blocking antibody or small inhibitory molecules was cytotoxic in multiple myeloma cells *in vitro* and in xenograft models (Trudel *et al*, 2006; Xin *et al*, 2006). In bladder cancer cells, knockdown of FGFR3 was achieved by short hairpin RNA constructs, leading to decreased proliferation, reduced clonogenicity and soft agar growth (Tomlinson *et al*, 2007). Our results confirm the strong anti-tumourigenic effect *in vivo* due to disruption of FGFR3 signalling in colorectal cancer. Infection of SW480 colon carcinoma cells with a KD3c-expressing adenovirus reduced tumourigenicity in a SCID mouse model by about 40% by both inhibition of proliferation and increased incidence of apoptosis.

Using *in vitro* models, reduction of colony formation and clonogenicity by KD3c could be shown. At higher cell densities, the dominant-negative construct reduced viability and induced apoptosis by activating caspase 3 by a non-mitochondrial pathway, as indicated by a stable status of mitochondrial membrane potential (data not shown). An additional effect of KD3c mutant on other FGFRs by heterodimerisation cannot be ruled out at this point. However, growth inhibition and induction of apoptosis could also be achieved by FGFR3-IIIc but not by FGFR3-IIIb knockdown, demonstrating that the IIIc splice variant is actually crucial for malignant growth and survival. Even though the *in vitro* effects may be relatively minor in some cases, together they are believed to synergistically cooperate in the anti-tumourigenic effect observed *in vivo*. As, for example, blocking of FGFR3 disrupts growth factor signalling and colony formation, both these mechanisms may cooperate in inducing apoptosis by loss of colony formation (Gilmore, 2005).

Assessment of FGFR-targeting strategies in tumour therapy has to take into account its effect on cell migration, which constitutes an important mechanism of invasion and metastasis (Dignass *et al*, 1994; Antoine *et al*, 2006). In neurons, FGFR1 associates with cell adhesion molecules, most importantly N-CAM, to stimulate neurite outgrowth and migration (Ronn *et al*, 2000). A similar configuration has been observed between FGFR4, N-CAM and N-cadherin in tumour cell migration (Cavallaro *et al*, 2001), providing a mechanistic explanation of the impact FGFR4 has on several tumour types (Stadler *et al*, 2006; Streit *et al*, 2006). Here, we demonstrate for the first time a major impact of FGFR3 on the migration of colon tumour cells. Although transfection of WT3c conferred sensitivity to FGF18-induced tumour cell migration, the dominant-negative KD3 virus reduced cell migration in both SW480 and Caco2 cells, but still permitted migration induced by 10% FCS. Together, these results indicate that autocrine stimulation by FGF18 is one, but not the only, mechanism inducing tumour cell migration. This indicates involvement of migratory signals unrelated to FGFRs, for example, c-met, which are also common in colorectal cancer (Otte *et al*, 2000).

In summary, our data demonstrate that FGFR3-IIIc exerts oncogenic effects in colorectal cancer cells by promoting *in vitro* tumour cell growth, survival, migration and responsiveness to oncogenic FGF ligands such as FGF18. Moreover, disruption of FGFR3-IIIc signalling distinctly attenuated colorectal cancer xenograft growth in SCID mice. This makes FGFR3-IIIc a promising candidate target for therapeutic interventions in colorectal cancer.

## Figures and Tables

**Table 1:** FGFR3 subtype expression and staging in colorectal tumour specimen

**Stage**	**Total number of specimen (*n*)**	**Specimen with increasing IIIc/IIIb ratio**	**% of total**	**Different from stage I (*P*-value)**
I	5	0	0	
II	26	12	43	0.0684 (NS)
III	15	9	60	0.0298 (^*^)
IV	6	4	67	0.0455 (^*^)

Abbreviation: FGFR3=fibroblast growth factor receptor 3; NS, not significant.

In all, 52 tumour specimens were classified according to the shift in the IIIc/IIIb ratio in comparison with the adjacent normal mucosa. Differences were assessed from contingency tables by Fisher's exact test.
