# Supplementary material for: Fibroblast growth factor receptor 3-IIIc mediates colorectal cancer growth and migration
Source: Br J Cancer. 2010 Mar 16;102(7):1145–56. doi: 10.1038/sj.bjc.6605596 (PMC2853090; doi:10.1038/sj.bjc.6605596)
Supplement: Supplementary Figure [file 6605596x1.ppt]

## Slide 1
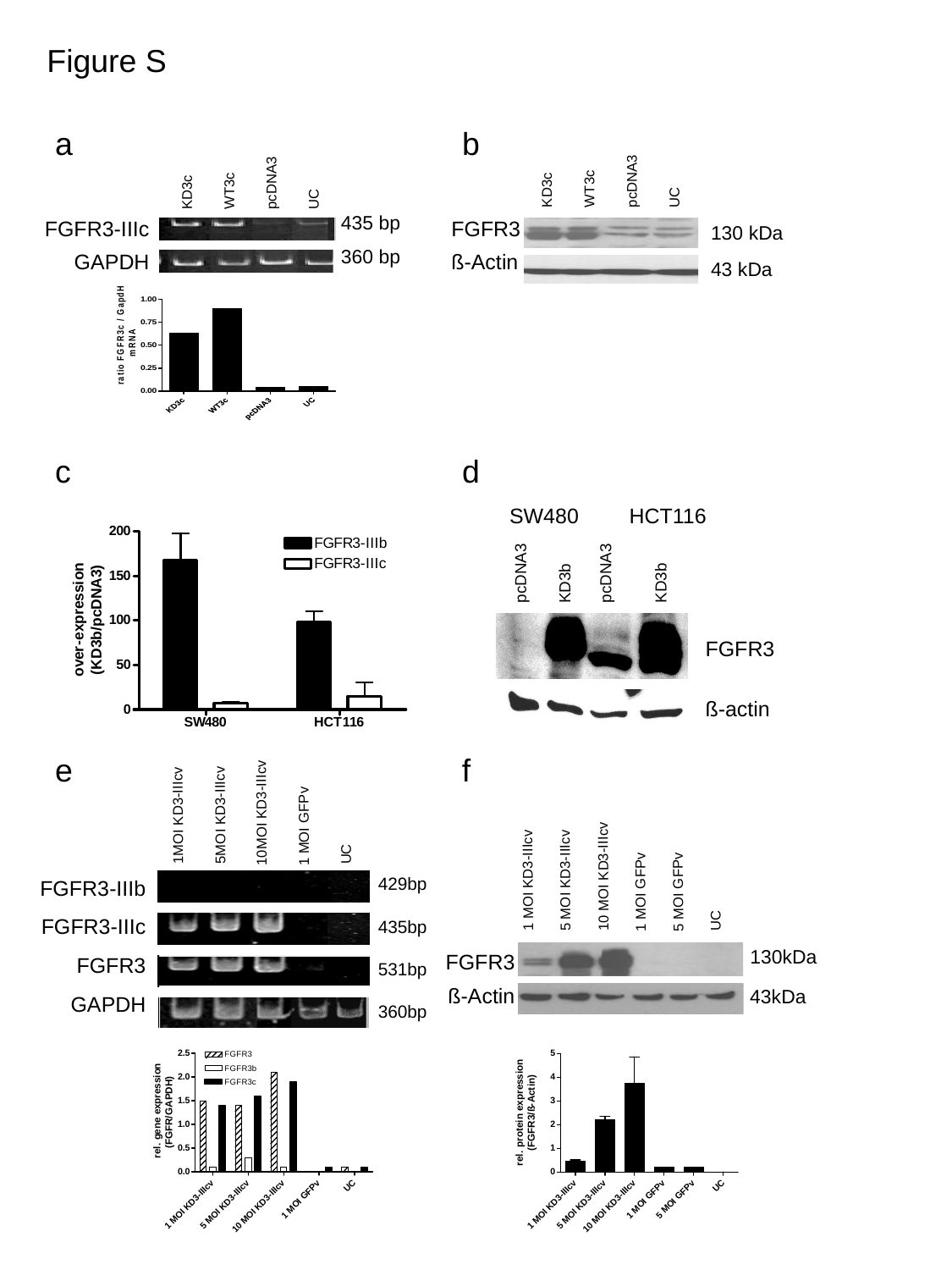

Figure S
a
b
KD3c
WT3c
pcDNA3
UC
FGFR3
ß-Actin
130 kDa
KD3c
WT3c
pcDNA3
UC
FGFR3-IIIc
GAPDH
435 bp
360 bp
43 kDa
c
d
SW480
HCT116
KD3b
KD3b
pcDNA3
pcDNA3
FGFR3
ß-actin
5MOI KD3-IIIcv
1MOI KD3-IIIcv
UC
1 MOI GFPv
10MOI KD3-IIIcv
 429bp
 435bp
 531bp
 360bp
FGFR3-IIIb
FGFR3-IIIc
FGFR3
GAPDH
e
f
UC
1 MOI KD3-IIIcv
5 MOI KD3-IIIcv
10 MOI KD3-IIIcv
1 MOI GFPv
5 MOI GFPv
130kDa
FGFR3
ß-Actin
43kDa
