# Supplementary material for: Fibroblast growth factor receptor 3-IIIc mediates colorectal cancer growth and migration
Source: Br J Cancer. 2010 Mar 16;102(7):1145–56. doi: 10.1038/sj.bjc.6605596 (PMC2853090; doi:10.1038/sj.bjc.6605596)
Supplement: Supplementary Information [file 6605596x2.doc]

Supplemental Material 1

**Methododical Details**

Standard RT-PCR conditions

| **gene** | **sense** | **anti-sense** |
| --- | --- | --- |
| total FGFR3 | 5’-AACGGCAGGGAGTTCCGCGGC-3’ | 5’-GTCAGCCTCCACCAGCTCCTC-3’ |
| FGFR3-IIIb | 5’-AACGGCAGGGAGTTCCGCGGC-3’ | 5’-CCCGTCCCGCTCCGACACATTG-3’ |
| FGFR3-IIIc | 5’-AACGGCAGGGAGTTCCGCGGC-3’ | 5’-CCCGGCGTCCTCAAAGGTG-3’ |
| GAPDH | 5´-cgggaagcttgtgatcaaTgg-3´ | 5´-ggcagtgatggcatgGactg-3´ |

Cycles of 40 seconds denaturation at 94°C, 40 seconds annealing at 56°C and 40 seconds extension at 72°C. Amplifications were done for 40 and 23 cycles in case of FGFR and GAPDH, respectively. PCR products were separated on 6% acrylamide gels. Bands were stained with ethidium bromide and quantified using a GelDoc system (Biorad, Hercules, CA) and Image Quant 5.0 (GE Healthcare, Piscataway, NJ) software.

**Antibodies for Western Blot**

| **Target** | **Company** | **Code** | **Dilution** |
| --- | --- | --- | --- |
| FGFR3 | Santa Cruz **Biotechnology, Inc.**, CA | sc-123 | 1:250 |
| phospho-S6 | Cell signalling, Boston, MA | #2215 | 1:1000 |
| S6 Ribosomal Protein |  | #2212, | 1:1000 |
| phospho-ERK1/2 | Cell signalling | #9101 | 1:1000 |
| ERK1/2 | upstate, Lake Placid, NY | #06-182 | 1:5000 |
| caspase 3 | Santa Cruz | H277 | 1:1000 |
| ß-actin | SIGMA, Saint Louis, MO | AC-15 | 1:5000 |

siRNA Sequences for FGFR3 knock down

| Oligo Name | Sequence | Ambion ID# |
| --- | --- | --- |
| FGFR3-IIIc 1 | AAACUUGUUCUCCACGACGca | s228721 |
| FGFR3-IIIc 2 | UUCUCCACGCAGGUGUag | s228722 |
| FGFR3-IIIb 1 | ACGCCUAUGAAAUUGGUGGct | s237345 |
| FGFR3-IIIb 2 | AUUGGUGGCUCGACAGAGGta | s237346 |

Supplemental Material 2

**FGFR3 overexpressing cell line models**

SW480 and HCT116 cells were used to create cell line models stably expressing FGFR3 by electroporation of either wild type (WT3c) or dominant-negative, kinase-dead (KD3c) cDNAs of FGFR3-IIIc or of a dominant-negative, kinase-dead forms of FGFR3-IIIb (KDb). Selection of clone pools stably expressing the transgenes was done with G418. Overexpression of FGFR3 in stable populations was checked by RT-PCR using IIIc- or IIIb-specific primers on mRNA (figure S a,c) and with Western-blot analysis on protein level (figure S b,d). From Caco2 cells stable transfectants could not be obtained. Furthermore, SW480 cells overexpressing KD3c had an extremely limited growth potential. Consequently, transient expression of dominant-negative FGFR3 was also achieved from an adenoviral construct expressing KD3 (KD3-IIIcv, figure S e,f). A GFP-tagged virus (GFPv), an untagged control virus (Cv), and uninfected parental SW480 cells were used for comparison. Splice variant IIIb mRNA was not influenced by ectopic expression of FGFR3-IIIc.

*Figure S:*

(a,b) In WT3c and KD3c transfectants overexpression of FGFR3-IIIc mRNA and total FGFR3 protein was demonstrated in SW480 cells stably expressing WT3c, KD3c or empty vector (pcDNA3) by standard RT-PCR (a) and Western-blot (b).

(c,d) In KD3b transfectants overexpression of FGFR3-IIIb was demonstrated by real-time RT-PCR, while FGFR3-IIIc expression was little affected (c) and by Western blot (d)

(e,f) SW480 cells were infected with adenoviral constructs expressing either dominant-negative KD3-IIIcv or GFPv. Expression of the transfected gene was demonstrated by standard RT-PCR (c) and Western-blot (d). For discrimination of the IIIc and IIIb splice variants of FGFR3 specific primers were used, recognizing either one or both splice variants (c). (c, lower panel). Western blotting was performed with a polyclonal antibody that recognizes both splice variants (d, upper panel).
